# Case Report: HGF and NF1 mutations as putative bypass mechanisms of MET inhibitor resistance in hepatocellular carcinoma: a case study

**DOI:** 10.3389/fphar.2025.1659463

**Published:** 2025-11-05

**Authors:** Zhitao Chen, Shan Luo, Yangjun Gu, Qiyong Li

**Affiliations:** ^1^ Department of Hepatobiliary Surgery, Shulan (Hangzhou) Hospital Affiliated to Zhejiang Shuren University Shulan International Medical College, Hangzhou, China; ^2^ Gastroenterology and Colorectal Oncology, The Third People’s Hospital of Zhengzhou, Zhengzhou, China

**Keywords:** hepatocellular carcinoma, MET amplification, crizotinib resistance, molecular resistance mechanisms, HGF and NF1 mutations

## Abstract

**Background:**

Hepatocellular carcinoma (HCC) is a highly aggressive liver cancer with poor prognosis, often associated with resistance to treatment. MET amplification has been identified as a potential therapeutic target, but resistance to MET inhibitors, such as crizotinib, remains a significant challenge. This study aims to explore the molecular mechanisms underlying resistance to MET inhibitors in MET-amplified HCC.

**Methods:**

We present a case of advanced HCC in a patient with MET amplification treated with crizotinib. After initial tumor regression, disease progression occurred. Genetic analysis using next-generation sequencing (NGS) was performed on biopsy samples taken before and after progression to identify mutations associated with resistance.

**Results:**

NGS revealed the loss of MET amplification and identified HGF and NF1 mutations as potential bypass mechanisms. Specifically, a missense mutation in HGF (p.G401A) was observed, which may enhance ligand-receptor binding, while an NF1 mutation (p.M546L) may permit sustained MAPK and PI3K activation despite MET inhibition. These observations are preliminary and require validation in larger patient cohorts.

**Conclusion:**

Our findings suggest that acquired resistance to MET inhibitors in MET-amplified HCC may involve clonal evolution and activation of compensatory signaling pathways. These insights highlight the need for dynamic molecular surveillance and the development of strategies targeting multiple pathways to overcome resistance and improve patient outcomes.

## Introduction

Hepatocellular carcinoma (HCC), the most frequent primary liver cancer, ranks sixth in global cancer incidence and is the fourth leading cause of cancer-related mortality worldwide (Siegel et al., 2023; Vogel et al., 2022). Owing to the typically late-stage diagnosis, patients with advanced HCC have a median survival of just 6–20 months (Anstee et al., 2019). In the United States, the current 5-year survival rate remains as low as 10% (Disoma et al., 2025). HCC presents significant global treatment challenges due to its pronounced heterogeneity and the limitations in prevention and early detection. Nonetheless, recent advances in systemic therapies—such as tyrosine kinase inhibitors (TKIs), immune checkpoint inhibitors (ICIs), and monoclonal antibodies—have led to improvements in both overall survival and quality of life for patients with advanced disease (Liu et al., 2023). However, these therapies typically prolong survival by only a few months, largely due to drug resistance and treatment-related toxicity (Chen et al., 2019). This highlights the urgent need to uncover the underlying mechanisms of resistance and identify novel therapeutic targets and agents.

Advances in next-generation sequencing and drug development have led to the identification of multiple proteins as promising therapeutic targets in HCC (Ran et al., 2025; Chen et al., 2021). The c-Met (MET) proto-oncogene, initially discovered as a tpr-met fusion in a chemically transformed human osteosarcoma cell line, encodes the receptor for hepatocyte growth factor (HGF) (Gherardi et al., 2012; Goyal et al., 2013). In the classic HGF/c-MET signaling cascade, HGF binding induces receptor dimerization and autophosphorylation at tyrosine residues in the c-MET C-terminal domain, subsequently activating downstream pathways such as MAPK, PI3K, and Rac1-Cdc42 (Gherardi et al., 2012). MET alterations act as oncogenic drivers in various cancers, arising either *de novo* or as acquired resistance to prior treatments (Bradley et al., 2017; Dagogo-Jack et al., 2020; Guo et al., 2020; Cerqua et al., 2025). Multiple MET-TKIs are currently used to target MET-altered tumors, with varying efficacy depending on the specific alteration (Drilon et al., 2020; Landi et al., 2019; Wolf et al., 2020). These inhibitors are classified as type I or type II. Type I TKIs, which compete with ATP and bind to the active (DFG-in) conformation of MET, are further divided into type Ia and type Ib (Fujino et al., 2019; Roskoski, 2016; Altintas and Comoglio, 2023). In contrast, type II TKIs target the inactive (DFG-out) form by extending into a hydrophobic pocket adjacent to the ATP-binding site (Fujino et al., 2019; Engstrom et al., 2017). In HCC, c-MET is frequently overexpressed, promoting tumor growth and angiogenesis, and is associated with poor prognosis (Gherardi et al., 2012; Goyal et al., 2013). Although the link between MET-dependence biomarkers and MET inhibitor response in HCC remains unclear, case reports suggest that HCC patients with MET amplification (METamp) may respond to MET-targeted therapies (Chen et al., 2021; Yan et al., 2023; Sang et al., 2023; Gu et al., 2023). However, in most patients, tumors eventually progress after a period of sustained partial response to effective therapy, likely due to the development of resistance to MET inhibitors (Altintas et al., 2023). The molecular basis of resistance to MET inhibitors, particularly in cases involving MET amplification, remains largely unclear. Beyond genomic alterations, recent work indicates that MET output can also be sustained by non-genetic, stress-adaptive programs; for example, activation of the integrated stress response (ISR) can enhance MET translation via upstream open reading frames in the 5′-UTR, increasing MET protein under hypoxia, nutrient deprivation, irradiation, or chemotherapy (Cerqua et al., 2025). Such ISR-driven translational control offers a mechanistic basis for maintenance of MET signaling under therapeutic pressure, potentially contributing to adaptive resistance even when MET copy number wanes.

We previously reported a case of HCC that achieved a favorable partial response following treatment with crizotinib, detailing the patient’s clinical background, treatment course, genomic findings, and therapeutic outcomes (Gu et al., 2023). Unfortunately, after a prolonged period of disease control, the tumor progressed again, suggesting acquired resistance to MET inhibitors. A repeat tumor biopsy was performed for genetic analysis, and we further explored the potential mechanisms underlying MET inhibitor resistance in this case.

## Case presentation

**FIGURE 1:**
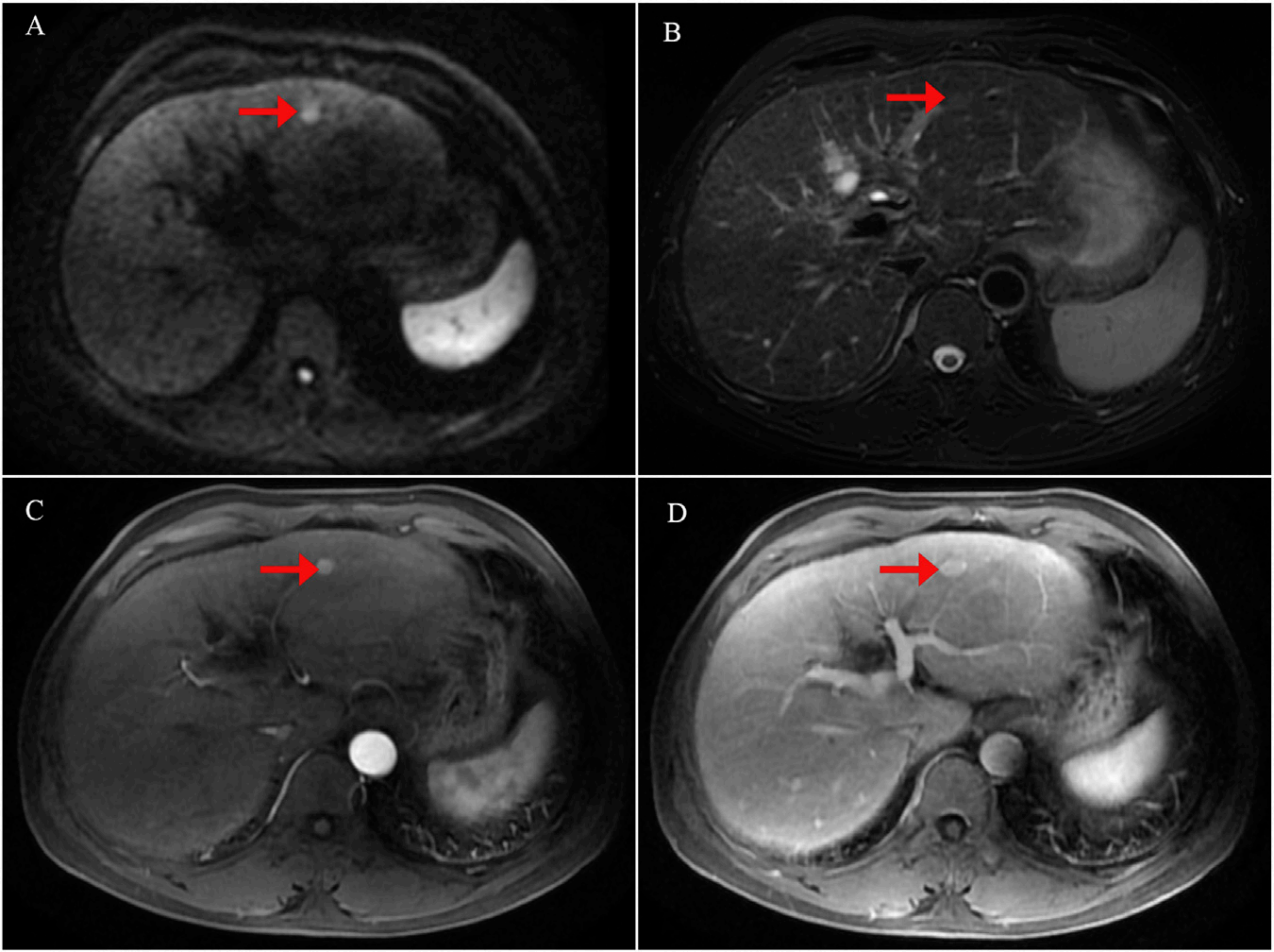
Initial contrast-enhanced liver magnetic resonance imaging (MRI) findings at the time of diagnosis. **(A)** Diffusion-weighted imaging (DWI) showing a focal area of restricted diffusion in the liver. **(B)** T2-weighted image demonstrating corresponding hyperintensity in the lesion. **(C)** Arterial phase of contrast-enhanced MRI revealing early enhancement of the lesion. **(D)** Venous phase image showing washout or delayed enhancement characteristics.

**FIGURE 2:**
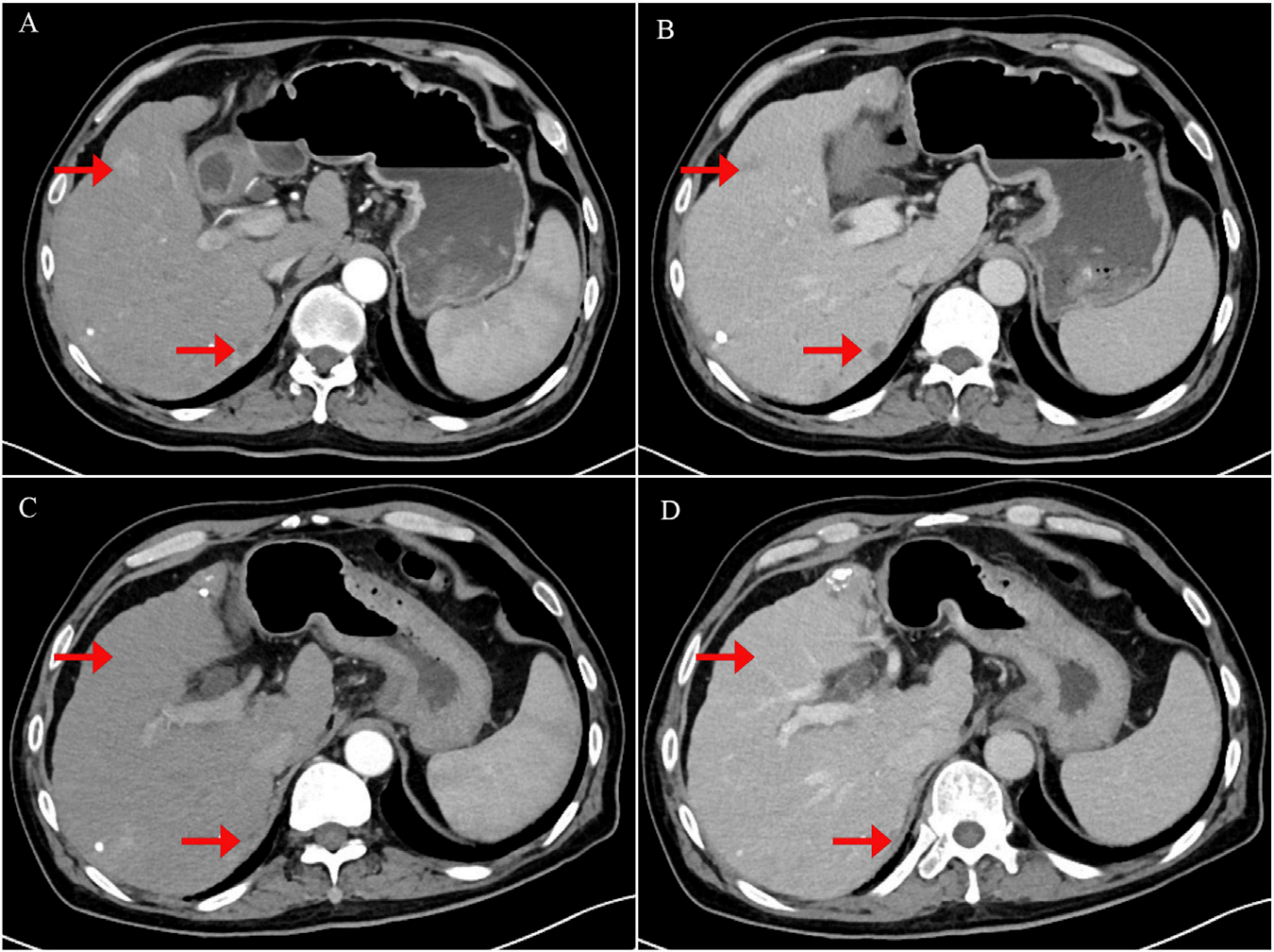
Contrast-enhanced abdominal computerized tomography (CT) scan images showing tumor recurrence and treatment response following crizotinib therapy. **(A)** Arterial phase image demonstrating a strongly enhancing nodule consistent with tumor recurrence. **(B)** Portal venous phase image showing persistent but slightly decreased enhancement of the lesion. **(C)** Arterial phase image obtained 1 month after initiation of crizotinib therapy, showing tumor shrinkage and reduced arterial enhancement of the nodule. **(D)** Portal venous phase post-treatment image demonstrating further tumor shrinkage with near-complete resolution of nodule enhancement.

A 56-year-old male with a more than 20-year history of chronic hepatitis B virus infection and liver cirrhosis was diagnosed with HCC in May 2019. The diagnosis was based on an elevated serum alpha-fetoprotein (AFP) level of 51 ng/mL and characteristic imaging findings that revealed a hepatic mass (Figure 1). The patient underwent a laparoscopic left lateral lobectomy, with the pathological diagnosis confirming moderately to highly differentiated HCC. The pathological findings were previously reported in an earlier publication (Gu et al.). In October 2020, local recurrence was detected, and the patient received radiofrequency ablation (RFA). Despite receiving multiple systemic therapies—initially lenvatinib plus camrelizumab, followed by sorafenib, and subsequently regorafenib in combination with sintilimab—as well as various local interventions including transcatheter arterial chemoembolization (TACE) and stereotactic body radiotherapy (SBRT), the disease progressed. Notably, vertebral metastases led to spinal cord compression and paraplegia. Given the advanced progression, next-generation sequencing (NGS) was performed and revealed MET gene amplification with a copy number of 30.2 (Table 1). Consequently, crizotinib was initiated at a dosage of 200 mg/day on 24 January 2022. This resulted in significant clinical and radiological improvement, including marked tumor regression, normalization of AFP and protein induced by vitamin K absence-II (PIVKA-II), and stable hepatic and renal function. Subsequent imaging demonstrated substantial reduction of tumor burden, with complete remission of certain lesions (Figure 2).

**TABLE 1:** Comprehensive genomic profiling of the initial biopsy from this hepatocellular carcinoma (HCC) patient identified multiple somatic alterations with potential clinical relevance.

Gene name	Mutation type	Amino acid change	Mutation location	Mutation frequency
ARAP3	2716G>T	G906C	exon19	19%
SYNE1	12,362_12363delinsGT	K4121S	exon76	57%
RELB	218C>G	P73R	exon3	50%
SPTA1	3,188 + 5G>A	—	intron22	52%
MDM4	512-4A>G	—	intron7	44%
DNMT3A	1015-3C>T	—	intron8	48%
TET3	1207G>T	A403S	exon4	48%
MLH1	1,039-8_1039-7insTTA	—	intron11	6%
FAT1	1519C>T	H507Y	exon2	46%
TRIO	31_36dup	P11_A12dup	exon1	29%
POLB	1002C>A	S334R	exon14	62%
NPAT	2692A>T	T898S	exon13	48%
SRGAP1	1658T>C	I553T	exon14	49%
DIS3	1978A>G	N660D	exon16	41%
MYH11	3848C>T	A1283V	exon29	10%
SERPINB4	837T>A	C279*	exon8	49%
BCORL1	26696G>A	R890Q	exon3	97%
MET	Rearrangement	—	intergenic/MET	30.2

*ARAP3* ArfGAP, with RhoGAP, domain, ankyrin repeat and PH, domain 3, *SYNE1* Spectrin repeat containing nuclear envelope protein 1, *RELB* RELB, proto-oncogene; NF-κB, subunit, *SPTA1* spectrin alpha, erythrocytic 1, *MDM4* MDM4 regulator of p53, *DNMT3A* DNA, methyltransferase 3 alpha, *TET3* Tet methylcytosine dioxygenase 3, *MLH1* MutL homolog 1, *FAT1* FAT, atypical cadherin 1; *TRIO*, Trio rho guanine nucleotide exchange factor, *POLB* DNA, polymerase beta; *NPAT*, nuclear protein, coactivator of histone transcription, *SRGAP1* SLIT-ROBO, Rho GTPase, activating protein 1, *DIS3* DIS3 homolog, exosome endoribonuclease and 3′-5′ exonuclease, *MYH11* Myosin heavy chain 11, *SERPINB4* Serpin family B member 4, *BCORL1* BCL6 corepressor like 1, *MET* MET, proto-oncogene, receptor tyrosine kinase.

**FIGURE 3:**
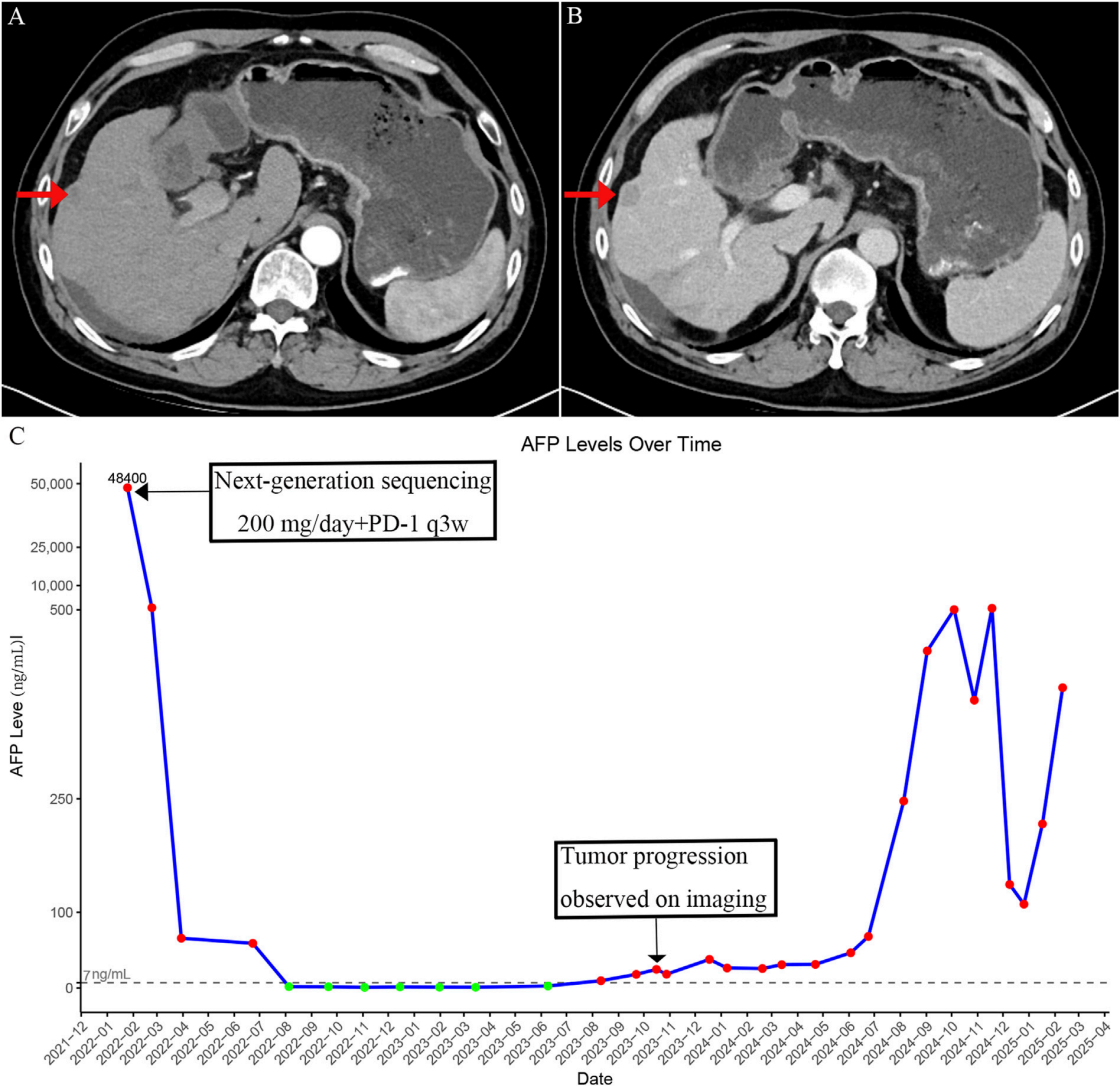
Imaging findings and serum alpha-fetoprotein (AFP) changes after development of resistance to crizotinib. **(A)** Arterial phase contrast-enhanced CT image showing intrahepatic recurrent nodules after acquired resistance to crizotinib. **(B)** Portal venous phase image showing decreased enhancement compared to the arterial phase in the recurrent intrahepatic lesions. **(C)** Time-course of serum AFP levels showing an initial decrease following crizotinib treatment, followed by a rise corresponding to disease progression after acquired resistance.

Unfortunately, after approximately 18 months of disease stability, a gradual and sustained increase in AFP was observed, eventually exceeding the normal range by August 2023 (Figure 3C). Imaging confirmed the emergence of new intrahepatic lesions (Figures 3A,B). In February 2025, a biopsy of the new hepatic lesion was performed, and NGS was repeated. However, no actionable mutations were identified (Table 2). Literature review suggests that resistance to MET-TKIs may be associated with specific mutations. MET-TKIs are classified into type I and type II inhibitors based on their binding modes to the ATP-binding pocket. It has been reported that mutations such as MET Y1230H confer resistance to type I MET-TKIs—including crizotinib, capmatinib, and tepotinib—but retain sensitivity to type II MET-TKIs, such as glesatinib and cabozantinib (Engstrom et al., 2017). Switching from a type I to a type II MET-TKI has been proposed as a strategy to overcome acquired resistance, especially in non-small cell lung cancer (NSCLC) (Cai et al., 2021). Despite the theoretical benefit, the patient was unable to afford cabozantinib due to its high cost and declined this treatment option. Subsequent treatments—including additional sessions of TACE and RFA, as well as changes in systemic therapies—yielded only temporary reductions in AFP levels, which quickly rebounded (Figure 3C). As of the latest follow-up, the patient remains alive but with persistent and uncontrolled disease activity. The most recent AFP level measured was 568 ng/mL.

**TABLE 2:** The next-generation sequencing (NGS) results from the second biopsy of the current hepatocellular carcinoma (HCC) patient revealed a set of gene alterations associated with disease progression and therapeutic resistance.

Gene name	Functional region	Mutation type	Amino acid change	Mutation frequency
ARID1A	exon 19	c.5079delC	p.L1694Cfs*9	53.87%
TP53	exon 4	c.G375T	p.T125T	53.37%
TERT	exon 1	c.-124C>T	—	41.40%
TERT	exon 1	c.-146C>T	—	41.40%
STAG2	exon 27	c.C2740T	p.Q914*	1.57%
HGF	exon 10	c.G1202C	p.G401A	36.74%
PMS1	exon 5	c.A575T	p.H192L	36.60%
NCOR1	exon 30	c.A3947G	p.K1316R	35.74%
PALB2	exon 4	c.T707C	p.F236S	32.16%
NF1	exon 14	c.A1636T	p.M546L	29.14%
EPHA2	exon 3	c.T562C	p.C188R	27.08%
FLT1	exon 21	c.G2878A	p.V960I	12.33%

*ARID1A*, AT-rich interaction domain 1A; *TP53,* Tumor protein p53; *TERT*, telomerase reverse transcriptase; *STAG2,* Stromal antigen 2; *HGF*, hepatocyte growth factor, *PMS1,* PMS1 homolog 1, mismatch repair system component; *NCOR1,* Nuclear receptor corepressor 1; *PALB2,* Partner and localizer of BRCA2; *NF1* Neurofibromin 1; *EPHA2* EPH, receptor A2; FLT1, Fms-related receptor tyrosine kinase 1 (VEGFR-1).

## Discussion

This case highlights acquired resistance to crizotinib in MET-amplified HCC documented with dual-timepoint tissue NGS. After a durable clinical and radiographic response, the progression biopsy demonstrated loss of MET amplification together with emergent HGF p.G401A and NF1 p.M546L, pointing to ligand reactivation and RAS/MAPK bypass as hypothesis-generating routes of escape in this tumor. To our knowledge, this is the first HCC report to longitudinally track these genomic shifts before and after crizotinib within the same patient, underscoring the need for further investigation into resistance mechanisms and alternative therapeutic approaches.

The MET proto-oncogene was initially identified in human osteosarcoma cells following exposure to N-methyl-N′-nitrosoguanidine, leading to its name (Cooper et al., 1984). It is located on chromosome 7q31.2 and spans approximately 126,191 base pairs. MET is a single-pass transmembrane receptor comprising extracellular, transmembrane, juxtamembrane, and intracellular tyrosine kinase domains. Its extracellular region binds HGF and includes a semaphorin (SEMA) domain, along with plexin-semaphorin-integrin (PSI) and immunoglobulin-like plexin transcription factor (IPT) domains (Ferracini et al., 2000). By binding to HGF, MET transmits signals from the extracellular matrix to the cytoplasm, activating pathways such as PI3K-AKT, Ras-Raf-ERK/MAPK, STAT-JNK, and SRC-FAK (Yang et al., 2022). These cascades regulate key biological processes, including cytoskeletal remodeling, embryogenesis, tissue repair, and organ regeneration, while also playing a central role in controlling cell proliferation, differentiation, and migration (Yang et al., 2022; Delitto et al., 2014). Multiple MET alterations—including exon 14 skipping (METex14), kinase domain mutations, amplification, and fusions—contribute to NSCLC development and EGFR-TKI resistance (Remon et al., 2023). Among these, METex14 skipping and amplification are especially significant for driving oncogenesis (Lee et al., 2021). While MET overexpression occurs in approximately 27.9% of cases, it shows limited correlation with recurrence, survival, or MET amplification (Lee et al., 2013). Previous studies have shown that MET amplification occurs in only 1.7% of HCC cases (Kondo et al., 2013). Consistently, analysis of the TCGA PanCancer dataset revealed a similarly low frequency of MET amplification in HCC, observed in just 2.17% (8 out of 369 cases).

Crizotinib, a small-molecule MET inhibitor, is FDA-approved for lung cancer but has not yet received approval for use in HCC patients with MET amplification (Lin and Shaw, 2017). Both previous case reports and our patient with advanced HCC demonstrated promising responses to crizotinib in the setting of MET amplification (Yan et al., 2023; Gu et al.). TKI resistance remains a major obstacle to sustained responses across targeted therapies, including crizotinib in MET-amplified HCC, where most patients eventually experience disease progression. In this case, crizotinib was given for high MET copy number and achieved prolonged remission, but resistance and tumor progression occurred after 18 months. A second biopsy followed by NGS revealed potential resistance mechanisms in this patient. Therefore, this study aims to further explore the underlying mechanisms of resistance to crizotinib in MET-amplified HCC. Although our understanding of resistance patterns and underlying molecular mechanisms is still evolving, emerging evidence provides valuable insights to support future drug development and optimize the clinical use of MET inhibitors beyond crizotinib.

In this patient, initial NGS detected MET amplification as the main oncogenic driver, making the tumor responsive to crizotinib. The patient maintained a favorable response for about 18 months, but disease progression was later accompanied by loss of MET amplification on repeat NGS, indicating a molecular shift in the tumor. Similar findings have been observed in some lung cancer patients progressing on first- or third-generation EGFR-TKIs or crizotinib (Wang et al., 2020). We speculate that loss of MET amplification contributed to resistance and tumor relapse in MET-amplified HCC following crizotinib treatment. One likely explanation for this phenomenon is clonal evolution, driven by intratumoral heterogeneity and selective pressure from therapy (Greaves and Maley, 2012). Cancer cells adapt to treatment through evolutionary processes shaped by their inherent genetic diversity. This heterogeneity exists both within a single tumor and between different tumor sites in the same patient (Amirouchene-Angelozzi et al., 2017). Tumors consist of multiple subclones with distinct genetic and phenotypic profiles (Greaves and Maley, 2012; Amirouchene-Angelozzi et al., 2017). Initially, MET amplification dominated, but crizotinib selectively suppressed MET-dependent clones. Over time, MET-independent subclones—either pre-existing at low levels or newly emerging—expanded and eventually became dominant. As a result, MET amplification was no longer detectable, and tumor growth continued via alternative oncogenic pathways. Beyond genetic drivers, adaptive non-genetic programs can also sustain MET output under therapeutic pressure; for example, activation of the integrated stress response (ISR) enhances MET translation via 5′-UTR uORFs and maintains MET signaling under hypoxia, nutrient deprivation, irradiation, or chemotherapy (Cerqua et al., 2025). In parallel, an AKT/mTOR-dependent feedback engaged upon MET-TKI withdrawal can simultaneously increase MET synthesis and blunt PTP1B-mediated dephosphorylation, producing a pathway “flare/rebound” despite loss of amplification (Altintas et al., 2023).

We also identified a TP53 mutation (p.T125T) with a variant allele frequency (VAF) of 53.4%, indicating clonal dominance. Although synonymous mutations are generally regarded as silent, emerging evidence suggests that those occurring within splicing or regulatory regions may disrupt gene function (Sharma et al., 2019; Oelschlaeger, 2024). Notably, TP53 mutations with high VAFs in HCC are frequently linked to impaired p53 signaling, contributing to therapeutic resistance, increased tumor aggressiveness, and defective apoptotic responses (Vokes et al., 2022). The loss of functional p53 may enable tumor cells to evade crizotinib-induced cytotoxicity and persist under treatment pressure.

**FIGURE 4:**
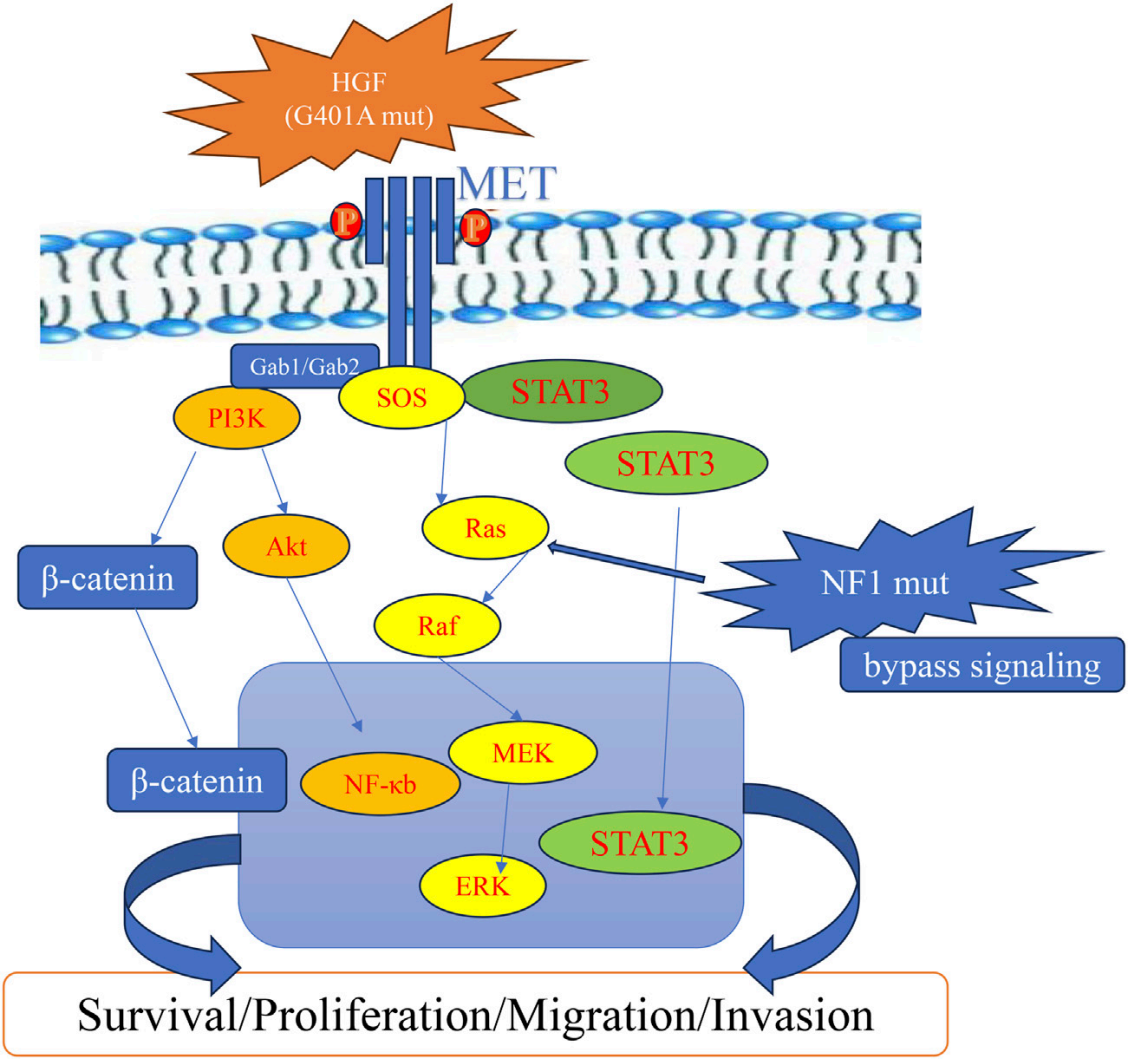
Schematic representation of a proposed HGF-MET pathway after acquired resistance to crizotinib, potentially involving HGF and NF1 alterations.

A missense mutation in *HGF* (p.G401A) with a VAF of 36.7% was identified as a prominent alteration in this patient’s post-progression genomic profile. The p.G401A substitution is located within or adjacent to the kringle domain of HGF, a region critical for receptor binding and ligand activation, as identified through UniProt database analysis (https://www.uniprot.org/). Although this specific variant has not been functionally characterized, we interpret p.G401A—located within/adjacent to the kringle region—as a possible activity-modulating substitution; given the single-patient design, we do not infer increased affinity or activation from our data (Figure 4). Moreover, the downstream effects of the HGF/MET signaling axis can vary depending on the microenvironmental context and the expression levels of ligands and receptors. In this light, we present the p.G401A variant as a hypothesis-generating observation, with ligand reactivation proposed as a plausible contributing factor, alongside NF1-mediated pathway bypass. Under normal conditions, MET activation in MET-amplified tumors is generally considered to occur independently of its ligand. However, increasing evidence suggests that elevated HGF levels—resulting from either stromal overexpression or genetic alterations—can overcome receptor blockade by reactivating MET signaling (Akli et al., 2025; Pennacchietti et al., 2014). This ligand-mediated reactivation has been identified as a critical mechanism contributing to resistance against MET-targeted therapies (Pennacchietti et al., 2014). Supporting this mechanism, preclinical studies in MET-amplified NSCLC and gastric cancer have demonstrated that high extracellular HGF concentrations markedly impair the efficacy of MET-TKIs (Pennacchietti et al., 2014). Notably, concurrent inhibition of active HGF can restore sensitivity to TKIs, emphasizing the therapeutic potential of dual-targeting strategies (Jones et al., 2024). These findings are further substantiated by experimental models showing that exogenous HGF stimulation reactivates MET and its downstream PI3K/AKT and MAPK signaling pathways, even in the presence of MET inhibitors such as crizotinib or tepotinib (Pennacchietti et al., 2014).

In addition to upstream ligand reactivation, the patient also harbored an NF1 p.M546L mutation. NF1 encodes neurofibromin, a tumor suppressor that negatively regulates RAS signaling by accelerating the hydrolysis of active RAS-GTP to inactive RAS-GDP (Anastasaki et al., 2022). Loss-of-function mutations in NF1 result in persistent RAS activation, leading to sustained signaling through the MAPK and PI3K/AKT pathways (Shapira et al., 2007). In the context of MET inhibition, this mechanism could constitute a bypass signaling route, whereby downstream effectors remain active despite upstream receptor blockade (Zhang et al., 2018) (Figure 4). Related patterns have been reported in MET-driven NSCLC, where NF1 alterations were associated with reduced sensitivity to crizotinib/tepotinib via sustained ERK signaling; however, direct evidence in HCC remains limited (Tao et al., 2020; Rotow et al., 2020).

Uncovering clonal evolution, *HGF* alterations, and compensatory signaling activation as resistance mechanisms offers critical insight into the therapeutic limitations faced by HCC patients harboring *MET* amplification. While agents like crizotinib have shown encouraging clinical activity in this molecular subset, the onset of acquired resistance remains a formidable challenge. To overcome this, therapeutic strategies that simultaneously inhibit both the kinase and juxtamembrane domains of MET may be necessary to suppress resistant subclones and enhance treatment durability. These findings also highlight the necessity of dynamic molecular surveillance during the course of therapy. The detection of resistance-associated events—such as attenuation or loss of *MET* amplification, activating mutations in *HGF*, and the activation of downstream escape pathways—may serve as early indicators of treatment failure and prompt timely therapeutic modification. Advancing our understanding of the molecular drivers behind *MET* inhibitor resistance is crucial for the development of innovative treatment paradigms designed to counteract resistance and prolong patient benefit.

This report has several limitations. First, it describes a single patient, which limits generalizability and precludes causal inference. Second, while dual-timepoint tissue sequencing is a strength, spatial heterogeneity and sampling from a new lesion at progression mean that the observed loss of MET amplification could reflect either true biological evolution under therapeutic pressure or outgrowth of a pre-existing MET-independent subclone. Third, we did not perform variant-specific functional assays for HGF p.G401A or NF1 p.M546L, nor splicing analyses for TP53 p.T125T; thus, any mechanistic role of these alterations in resistance should be regarded as hypothesis-generating. Finally, although we discuss non-genetic maintenance of MET signaling based on the literature cited, such adaptive mechanisms were not directly tested in this case. Validation in larger cohorts and experimental models will be required to determine the prevalence and functional impact of these events in MET-altered HCC.

## Conclusion

This single-patient case shows that acquired resistance to crizotinib in MET-amplified HCC can arise via two non-exclusive routes: attenuation or loss of the original MET driver and the emergence of putative bypass alterations (HGF p.G401A and NF1 p.M546L). The key clinical implication is that dynamic molecular monitoring—including planned re-biopsy and repeat NGS at progression—should be incorporated into routine care to detect such evolutionary shifts and guide timely therapeutic adjustment. While the variant-level inferences here are hypothesis-generating, longitudinal profiling provides practical value for considering alternative strategies or clinical-trial options. Larger cohorts and functional studies are needed to define the prevalence and actionability of these resistance trajectories in HCC.

## Data Availability

The datasets presented in this article are not readily available because of ethical and privacy restrictions. Requests to access the datasets should be directed to the corresponding author.
